# Identifying the content, functionalities, and features of a mobile application for contact lens wearers

**DOI:** 10.1186/s12911-022-01910-w

**Published:** 2022-06-21

**Authors:** Fatemeh Falahati-Marvast, Andrew D. Pucker, Fateme Alipour, Jamileh Farokhzadian, Leila Ahmadian

**Affiliations:** 1 Department of Health Information Sciences, Faculty of Management and Medical Information Sciences, Kerman University of Medical Sciences, Kerman, Iran; 2 Department of Optometry and Vision Science, School of Optometry, University of Alabama at Birmingham, Birmingham, AL USA; 3 Eye Research Center, Farabi Eye Hospital, Tehran University of Medical Sciences, Tehran, Iran; 4 Nursing Research Center, Kerman University of Medical Sciences, Kerman, Iran; 5 Department of Community Health Nursing, Razi Faculty of Nursing and Midwifery, Kerman University of Medical Sciences, Kerman, Iran

**Keywords:** Contact lenses, Contact lens wearers, Mobile application, Mobile health, Needs assessment, Qualitative research, User-Centered Design

## Abstract

**Background:**

Lack of attention to end-users' requirements and preferences may lead to the failure of health information technology (IT) interventions. Identifying users’ needs for designing a mobile application can lead to the development of an acceptable intervention. This study aimed to determine the requirements for designing a mobile application to educate and provide needed information to contact lens (CL) wearers.

**Methods:**

A qualitative study was conducted on 24 CL wearers and nine eye care practitioners from the three CL clinics in Iran. Data were collected through semi-structured interviews and analyzed using the conventional content analysis proposed by Lundman and Graneheim. Lincoln and Guba's criteria were used to ensure the trustworthiness of the data.

**Results:**

The three main categories that emerged from the interviews were mobile application content, mobile application functionalities, and mobile application features. Ten subcategories and 57 sub-subcategories were also identified. It was determined that mobile content should focus on advice and information for optimizing the CL wearing experience and training regarding the use of CLs. Entering information required for self-care, computational capability, interactivity, updates, and reminders were determined as needed functionalities. The participants recommended features for the structure and user interface of the mobile application and information presentation methods.

**Conclusion:**

This study identified the information needed to develop a mobile application for CL wearers. This also provides insights regarding required functionalities when applying IT interventions. These findings can be used by CL clinics, developers of health information systems, policymakers, and health planners to design better CL-related care and compliance interventions.

**Supplementary Information:**

The online version contains supplementary material available at 10.1186/s12911-022-01910-w.

## Background

Contact Lenses (CLs) are extensively used worldwide for both therapeutic and cosmetic purposes [1, 2]. With millions of CL wearers in the world [3], there needs to be adequate patient information that describes the proper CL application and removal, CL care regimen, and all other instructions needed to minimize and prevent CL complications [1, 4, 5]. Various complications with different incidence percent such as dry eye with 50% incidence, discomfort with 23–94%, and corneal staining with 54% can threaten eye health and lead to decreased wearing time or even discontinuation of CL wear [6].

Improper and inadequate information leads to complications that threaten eye health. Acquiring relevant information is an important aspect of CL care because having this information enables CL wearers to have greater control over their self-care and ocular health [2, 7].

The recommended CL information is typically provided by CL practitioners in a face-to-face meeting after CL prescription, and patients may have minimal training after their initial CL fit and training session [8–11]. Because of the CL wearer’s involvement in the process of applying and removing the CL, the stress associated with learning this new complex task, and the short duration of time usually associated with in-office training, CL wearers may not be able to fully understand and retain all the received information provided during the initial training session. Moreover, the high workload of practitioners may hinder them from consistently providing adequate CL care and compliance information [8, 12, 13]. This is highlighted by some previous studies that have shown that CL wearers have little knowledge related to CL care and compliance [1, 4, 5, 14, 15] and have various information needs in this field [16]. These researchers furthermore emphasized the need to move away from conventional teaching practices and toward new educational strategies that involve modern technology [5, 17–19].

Smartphone-based strategies are an area that provides a useful starting point for establishing a new educational approach for CL wearers [20]. Providing repeatable training and information via mobile devices, and interacting with patients through the mobile application has the potential to increase successful CLs wear and patient peace of mind. A mobile application tailored to the needs of CL wearers can increase the knowledge of CL wearers and potentially prevent CL-related complications.

By designing a mobile application according to the needs of CL wearers through a User-Centered Design (UCD) approach, the acceptance and effectiveness of the mobile application will be ensured. The UCD approach is an iterative process that typical end-users are incorporated in the design process [21].

To our knowledge, the field currently lacks a scientifically designed mobile application for CL wearers to help them to improve their self-care through appropriate and tailored interventions. Therefore, as part of the UCD process, information needs of CL wearers were determined in a previous study [16] and the present study qualitatively identified the requirements that should be considered when creating a mobile application for CL wearers to provide information and services tailored to their expectations.

This application can be useful for both new CL wearers who are new to the subject of CL and need training and repetition of information and past CL wearers. Both groups can use the information and services of the application to have the best lens care and prevent complications.

## Methods

### Design, setting, and participants

This study was conducted using a qualitative approach to explore the perspectives of CL wearers and practitioners regarding the development of a mobile application for CL wearers to provide required education and health care information. For maximum diversity, participants included CL wearers or their parents when applicable, ophthalmologists, and optometrists (an ophthalmologist is a medical doctor, and an optometrist is a care provider who provide primary vision care) from three CL clinics in Tehran, Iran. Two of these clinics were affiliated with Farabi and Noor ophthalmology hospitals. Farabi hospital is the only ophthalmology academic center affiliated to Tehran University of Medical Sciences. The third center was a well-known specialized private ophthalmology clinic that prescribes soft and corneal gas-permeable CLs. A purposive sampling method was applied to recruit the CL wearers. All practitioners working in the clinics including ophthalmologists and optometrists were interviewed for understanding requirements in the development of the mobile application. Neophytes (a person who is new in using CL) who received routine training, established CL wearers as defined by at least one month of CL wear, and past CL wearers were included. Parents of young patients were likewise included to understand the full needs of all CL wearers.

This study was accomplished according to the Consolidated Criteria for Reporting Qualitative Research (COREQ) [22]. We considered 30 out of 32 items of COREQ in our study. A checklist is provided in Additional file 1.

### Data collection

All interviews were conducted in a semi-structured, face-to-face format in a comfortable, private room by a member of the research team [FFM] (This researcher is a woman with a master's degree in medical informatics and 8 years of experience in this field) between July 2019 to September 2019. To collect the data, the interviewer introduced the study and its objectives. Data collection was continued until data saturation as defined by the time that new interviews produce only previously discovered data. A series of open-ended interview questions were designed, revised, and confirmed by the research team. All questions were administered in Persian and translated into English to present in this manuscript. The main questions are shown in Table 1. Every interview was recorded with a voice recorder and handwritten notes were taken when needed. Interview length ranged from 35 to 70 min. If participants required more time, a second interview session was scheduled.

**Table 1:** Interview topic guide

Interview questions
1. What is your opinion about the idea of using a mobile application for contact lens wear?
2. What expectations do CL wearers have from a mobile application to assist them with wearing and caring for contact lenses?
3. What issues should be considered if a mobile application will be designed for CL wearers?
4. What information would CL wearers like to receive from the mobile application?
5. How should a mobile application for CL wearers be designed to be acceptable and usable for them?
6. How can a mobile application help CL wearers?
7. How should a mobile application present information to CL wearers?

### Data analysis

This study used conventional content analysis. Data were analyzed as recommended by Graneheim and Lundman [23]. One researcher [FFM] listened to the recorded interviews and transcribed them verbatim. Entire transcripts were then read by two investigators [FFM, JF-PhD] for a comprehensive understanding of their content and defining the units of meaning and codes. Codes were defined as discovered concepts from interviews by analyzing words and sentences. Codes were compared with each other according to their similarities in terms of the meaning and concept and summarized under more comprehensive classifications of the data that named sub-subcategories [FFM, ADP-PhD, FA-MD, JF, LA-PhD]. Similarly, recognized sub-subcategories were grouped under subcategories [FFM, ADP, FA, JF, LA]. Main categories emerged from subcategories according to a common meaning [FFM, ADP, FA, JF, LA]. Despite the inductive nature of this method, a back-and-forth movement was carried out between the whole and parts of the text to obtain the most internal consistency and least external incompatibility. All researchers checked codes, sub-subcategories, subcategories, and main categories. Discrepancies were then resolved and a consensus was reached after discussion. The MAXQDA 10 software was employed for data management.

### Trustworthiness of data

Guba and Lincoln's criteria [23] were used for supporting processes of data trustworthiness including confirmability, dependability, credibility, and transferability. The investigators were engaged with each of the participants, when required comments or corrections were received from the participants regarding their transcripts. All recordings, documents, and notes were retained for future review. Frequent reporting sessions were held between members of the research team, and all team members commented on the codes, sub-subcategories, subcategories, and main categories before proceeding with the next development step.

The authors paid attention to reflexivity during the data collection and analysis to avoid biases associated with their own experiences and belief in this study. The first researcher [FFM] performed this process by not being actively involved in the interviews and not reacting to the leading questions and CL wearer's responses. Also, a research team from various professional disciplines collaborated in data analysis to complete each other and control the influence of personal opinion.

### Ethical considerations

This study was approved by the research ethics committee of Kerman University of Medical Sciences (Code: IR.KMU.REC.1400.254). At the request of the Ethical committee, the study was conducted in accordance with the Declaration of Helsinki and Ethics Publication on Committee (COPE). Informed consent was obtained from all participants or if participants are under 16 and/or illiterate people, from a parent and/or legal guardian before recording the interviews. During the research, voluntary participation, the anonymity of the participants, the confidentiality of the information, and the right to withdraw at a desired time were considered. Necessary permission and coordination were made with the clinic officials to conduct the study.

## Results

Thirty-three participants both male and female including 24 CL wearers, three ophthalmologists, and six optometrists (with Bachelor's and Master's academic degrees) were interviewed. Four participants asked for a second interview session. Details of participants are shown in Table 2. Some information and care services needs were identified jointly by CL wearers and practitioners, but some of them were described only by either CL wearers or practitioners (Table 3). Three main categories including mobile application content, mobile application functionalities, and mobile application features with 10 subcategories and 57 sub-subcategories were extracted from data analysis (Table 3). The main categories and subcategories with some exemplary quotes are shown in Table 4. We provided all quotes for each statement in Additional file 2.

**Table 2:** Participant demographics

Contact lens wearers		Eye care practitioners	
Characteristics	n (%)	Characteristics	n (%)
Age		Profession	
< 5^a^	2 (8.33)	Ophthalmologist	3 (33.33)
5–15^a^	2 (8.33)	Optometrist	6 (66.67)
16–25	3 (12.5)		
26–35	8 (33.34)		
36–45	7 (29.17)		
46–55	2 (8.33)		
Education		Work experience (year)	
Illiterate	3 (12.5)	< 5	1 (11.11)
Elementary school	1 (4.16)	5–15	5 (55.56)
High school	1 (4.16)	16–30	1 (11.11)
Diploma	5 (20.83)	31–45	2 (22.22)
Bachelor	7 (29.17)		
Master	7 (29.17)		
Lens type^b^		Work experience in CL clinic (year)	
Soft	5 (17.24)	< 5	1 (11.11)
Corneal RGP	20 (68.96)	5–15	5 (55.56)
Scleral RGP	4 (13.80)	16–30	1 (11.11)
Contact lens wear duration^b ^		31–45	2 (22.22)
Neophytes	7 (24.14)		
1–3 years	8 (27.59)		
> 3 years	14 (48.27)		

*RGP* rigid gas permeable

^a^The parents answered the questions

^b^Some participants experienced more than one type of lens

**Table 3:** Identified requirements for designing a mobile application for contact lens wearers

*1. Mobile application content*	*2. Mobile application functionalities*	*3. Mobile application features*
*1.1 Advice and precaution*	*2.1 Entering information required for self-care*	*3.1 The structure and user interface of the mobile application*
Basic information for CL wearers^c^	Entering details of practitioner’s visits (visit date, name of the clinic, name of practitioner, medications, instructions…)^c^	Easy to use^a^
CL hygiene information^c^	Entering name and type of CL^c^	Short and simple tutorials^a^
CL care information^c^	Entering CL parameters^a^	Attractive user interface^a^
CL complications information^c^	Entering manufacturing and expiry dates of CL^a^	Correct and reliable information^c^
Providing instructions for CL wearers for using the CL^b^	Entering start and end dates of CL use^a^	In agreement with the culture^a^
Presenting the results of new research regarding safety in using the CL^a^	Entering the hours of gradual use of the hard CL in the CL habit stage^c^	*3.2 Information presentation methods*
*1.2 Training*	Entering CL wearing hours during the day^c^	Text^a^
Training of CL insertion tailored to the type of CL^c^	Entering the time of day when the CL wearers feel discomfort^c^	Video^a^
Training of CL removal tailored to the type of CL^c^	Entering dates and times of pain while using the CL^a^	Animation^a^
Training of CL rinsing and disinfecting^c^	Entering complications and their occurrence time^c^	Voice^a^
Training of handwashing and disinfecting^c^	*2.2 Computational capability*	
Training of CL case rinsing and disinfecting^c^	Calculation of CL usage time over the day, week, and month^c^	
Specific training tailored to the target population^a^	Calculation of the time period of CL use and the start of uncomfortable feeling^b^	
*1.3 Supplementary information for better CL wearing*	Calculation of the total days of pain^a^	
Introducing CL accessories^c^	*2.3 CL wearers interaction with mobile application*	
Introducing common CL brands^c^	Asking questions from a practitioner^a^	
Introducing CL certified sales centers^c^	Creation of frequently asked questions list^a^	
Introducing information resources^a^	Rating the practitioner’s answers by CL wearers^a^	
Introducing new initiatives in the field of CL^a^	Expressing the concerns, opinions, and experiences of CL wearers^a^	
Introducing well-known centers with their address^c^	Rating the experiences and comments^a^	
	Comments on the CL wearers’ input^a^	
	Organizing and prioritizing CL wearers’ experiences and comments based on the ratings^a^	
	Searching answers and experiences^a^	
	Making appointments for next visits^a^	
	*2.4 Mobile application development*	
	Creation of a mobile application in different languages such as English in addition to Persian^a^	
	Mobile application updates according to new needs, suggestions, and feedback^a^	
	Development of the mobile application based on best available evidence^b^	
	*2.5 Reminders and alerts*	
	Setting up the reminders or alerts times^c^	
	Reminders of CL use^c^	
	Reminder for CL and CL case replacement time^c^	
	Reminder for visiting practitioner^c^	
	Alert for entering CL use information^c^	

*CL* contact lens

^a^Needs described only by CL wearers

^b^Needs described only by Practitioners

^c^Needs described jointly by CL wearers and practitioners

**Table 4:** Interview excerpts supporting key factors related to the content, functionalities, and features categories and their subcategories

Main categories along with subcategories	Example of interview excerpts
*1. Mobile application content*	
1.1 Advice and precaution	1.1 I need information on the type of lens, how to use the lens, why I am using it, how the lens works and interacts with the eye, how to prepare for the visit, and the necessary examinations to prescribe the lens. When I want more information about CL, practitioners are always busy, making it difficult to ask additional questions about CL. (CL wearer, male, 27 years old)
1.2 Training	1.2 CL wearers with varying educational levels, age groups, and underlying diseases, all receive the same training. What should I know about children's lenses if I need special training for my baby, such as how to put a lens for him? (CL wearer, male, 3 months of age)^a^
1.3 Supplementary information for better CL wearing	1.3 CL wearers should have access to information about the common brands of CL and lens solutions. "Tell us, what the good brands are?" my friends asked me. I talked to my friends about the lens brand. Some of my friends used low quality colored CL, resulting in red eyes. I recommended good brands to my friends. “Choose between not putting a lens and putting a good lens” I said. (CL wearer, female, 31 years old)
*2. Mobile application functionalities*	
2.1 Entering information required for self-care	2.1 CL wearers forget which clinic they went to and when they went, when they received the lens and when they changed the lens. CL wearers did not follow the practitioner’s instructions because they had forgotten many of instructions. For example, one of CL wearers received the lens a year ago and claims to have received it six months ago. (Optometrist, female, 30 years of work experience in CL Clinic)
2.2 Computational capability	2.2 When I ask the CL wearers how many hours a day they wear the CL, some of them have no idea. It is important for us to know how many hours a day and how many days a week CL wearers put in CL. CL wearers often reduce CL use hours due to discomfort. (Ophthalmologist, female, 10 years of work experience in CL Clinic)
2.3 CL wearers interaction with mobile application	2.3 I would like to have my questions answered. Include the possibility of questions and answers between CL wearers and practitioners in the application. Even if practitioners do not have the time to answer questions online and individually, they can see and answer the questions every few days. (CL wearer, male, 48 years old)
2.4 Mobile application development	2.4 If the application is presented in English in addition to Persian, more CL wearers will use it and more comments and problems will be expressed. CL wearer’s data can be used to develop the next version of application as well as to conduct new research to solve many CL wearers’ problems. (CL wearer, female, 36 years old)
2.5 Reminders and alerts	2.5 CL wearers usually forget the follow-up, when to consult a practitioner, when to change the CL, and so on. Some of them have a regular lifestyle and forget less, while others do not. The application should include a reminder. (Optometrist, female, 10 years of work experience in CL Clinic)
*3. Mobile application features*	
3.1 The structure and user interface of the mobile application	3.1 If I want an application, it must have additional features and value as well as an interesting design that I tend to use. I will not make use of it by only providing a list of information. (CL wearer, male, 24 years old)
3.2 Information presentation methods	3.2 The clinic gives us a brochure, but if our vision is poor, we cannot read it. One person is old, the other is illiterate, and both need someone to read the brochure to them. (CL wearer, male, 29 years old)

*CL* contact lens

^a^The characteristics are related to the CL wearer but their parents answered the questions

### Main Category 1: Mobile application content

Participants indicated that the contents of the mobile application should contain advice and precaution, training, and supplementary information for better CL use.

According to the participants, the mobile application should contain useful and basic information about the eyes, CLs and its types, advantages, and disadvantages of each CL type, CL application, CL lifespan, and reasons for using it, which will increase knowledge, reduce concern, and facilitate easier CL acceptance. Informing CL wearers of CL prescription examination will lead to better patients’ cooperation with the practitioner. Participants stated that gaining the necessary information before making an appointment with practitioners will reduce unnecessary visits and wasted time. Practitioners mentioned that providing information and instructions to CL wearers in various fields of CL such as use, care, and hygiene through the mobile application can reduce complications and improve compliance behaviors. Explaining the risks and complications of CLs can lead to more adherence to instructions. CL wearers believed that information about recent research and developments in the field of CL will motivate them to use CL. According to CL wearers, the results of research in various fields such as use, care, hygiene, design of new CLs, and ways to reduce complications can change their behaviors and help improve the use of CL.

CL wearers stated that after receiving their CLs, they engage in routine clinical training on the process of applying and removing their CLs. Also, due to the short time and high workload of the hospital-based clinic that they visited and the large number of patients undergoing CL training, they felt that they did not learn how to wear their CLs well initially. Alternatively, due to involvement in this process and having learning stress, the patients usually forget other information that was given to them at the training session, such as hand washing, disinfection, and practitioner’s recommendations.

The patients expressed that CL training in the form of multi-step text instructions were unhelpful, and they wanted to be provided with applicable, practical, and interactive training with the potential of repeatability. They also wanted to be provided specific training tailored to the target group (e.g., children, adults, patients with underlying diseases).

Parents stated that they need specific training in the use and care of CLs in children. They are worried about the future of their children's vision and believed that because children, especially infants, cannot express their feelings and problems, the responsibility of using CLs for children is more worrying for them. Parents wanted to get enough information and knowledge on various topics such as common problems that lead to the use of CLs in children, applying and removing CLs, and CL care.

CL wearers, especially beginners, wanted to be informed about well-known CL brands, delivery and sales centers of CLs and prescription and service centers of CL, and their location. Moreover, the patients tended to want to know about skilled and experienced practitioners to receive and use the CLs with more confidence and assurance. The number of experts and centers in the field of CL in Iran, especially hard CL, is low. Also, most of them are located in large cities. Because of these, most CL wearers, especially those who traveled from other cities to receive CLs, wanted to know about the CL practitioners and centers in their area of residence.

According to CL wearers, informational resources are important because they allow the patient to get more information. Knowing the innovations both in the CL and in its accessories, in addition to the possibility of using them, is associated with being better informed. This information also demonstrates to the patient that the CL manufacturers and researchers are considering CL wearers’ problems and striving to improve the products.

### Main Category 2: Mobile application functionalities

Practitioners said that most CL wearers do not know the answer to questions such as the type of CL, the starting date of use, the time of CL use during the day, and the time of complications occurrence. They also do not have a comprehensive understanding of CL wear. Alternatively, CL wearers wanted the mobile application to be able to document important data because many of them forget the information about their CL and the times associated with using them. Recording different information helps to track CL wearers’ behavior, and practitioners can receive information related to activity tracking and symptom tracking of CL wearers for better decisions.

CL wearers wished the mobile application, like a personal health record (PHR), providing information about their specific CL, as well as to calculate different time intervals of CL wear for them, so they could provide this information to practitioners and answers their questions better. The patients felt that this information would help practitioners make better CL-related decisions.

CL wearers were keen to be able to connect with other CL wearers and share their experiences, concerns, and CL-related challenges. Discussion forums bring together people undergoing the same condition. The patients expressed that listening to the experiences and opinions of others can give them a greater sense of calm because they realize that other CL wearers were having similar experiences. However, the accuracy of shared experiences was a concern stated by CL wearers, and they wanted an expert to investigate and verify the validity of these experiences. Another request of CL wearers was the ability to rate experiences and opinions and display experiences that received the highest score. CL wearers wanted to get in touch with experts in the CL field and be able to ask them questions and receive responses appropriate to their conditions, especially people who live in other cities and do not have access to a practitioner. The patients wanted the possibility of searching in asked questions and experiences of others to easily find intended content.

CL wearers wanted to be able to set reminders and receive alerts and notifications based on the information entered into the mobile application and the calculations. In their opinion, the importance of regular follow-up indicates the necessity of the practitioner's visit reminder. Also, even if they forget to enter the information, they suggested that notifications should be sent to them to remind them to enter the information. CL wearers wanted that the reminder should be designed in such a way that it is noticeable and not be overlooked.

The patients stated that it should be possible to update the mobile application based upon new feedback and needs and the best available evidence. The patients declared that a mobile application design with no updates would reduce CL wearers’ willingness to use it. The development of an English version of the mobile application as a common language in the world in addition to the local language (Persian) would cause more CL wearers to use it, which eventually would lead to getting more feedback and better development of the mobile application in the future. Meanwhile, recognizing more opinions, experiences, and problems of the CL wearers could lead to the development of new CLs and CL-related knowledge.

### Main Category 3: Mobile application features

CL wearers wanted the mobile application to be designed in a simple manner so that it can be used easily. The patients suggested applying large font, large buttons, and contrasting text for more simplicity and usability. Wearers stated that the existence of many menus and complexities will cause confusion in their use and unwillingness to utilize the mobile application. Also, the patients suggested using easy ways to enter information and set up reminders such as drop-down lists, toggle switches, date and time pickers, and checkboxes.

Participants tended to desire accurate and reliable information and simple and concise tutorials without complicated medical terminology in a simple and local language based on their culture through an appealing interface. Patients expressed the training and information be presented in the form of multimedia material such as video or short animations with audio and subtitle. CL wearers also preferred sound over text and preferred minimal text in the mobile application due to various reasons such as difficulty in reading small text and inability to read the text if they had low vision.

Generally, most of the preferences for the mobile application content were reported jointly by both the CL wearers and practitioners. Both groups wanted a CL mobile application to have functionalities that help patients in CL care and use. In addition, both groups thought that reminders should have a positive impact, but these reminders should be designed to include a good solution for ignoring warnings and alert fatigue. Moreover, different perspectives of both groups identified various types of information to enter and save into the mobile application. CL wearers wanted a CL mobile application that engages them and provides feedback. While the mobile application features are mostly designated by the CL wearers, the practitioners pointed out the correct and reliable information feature.

## Discussion

To our knowledge, this is the first study that has explored the viewpoints of CL wearers and eye care practitioners to understand the requirements for designing a mobile application for CL wearers. Based on this study’s findings, three main categories including mobile application content, mobile application functionalities, and mobile application features should be considered when developing a mobile-based intervention.

Our study findings showed that the CL wearers need to have knowledge regarding different aspects of the CL such as CL use and care, CL training regarding application and removal, rinsing and disinfecting of CLs, brands and certified sale center to perform CL care, and information to prevent complications, and preferred to develop the mobile application content based on the reported information needs. In agreement with our findings, various studies have determined that CL wearers have different information needs [16] and limited knowledge related to using and caring for CLs, potential complications of CLs, cleaning of CLs and cases, and hand hygiene [10, 15, 24, 25]. In line with our findings, several studies suggested the need for the development of training strategies and also providing enough information and theoretical and practical education for CL wearers to be knowledgeable about all aspects of the CL use and care [1, 2, 10, 14, 18, 25–28]. In addition, studies similar to our study emphasized that lack of information, misconceptions, insufficient available information resources, purchasing places on the internet, and unclear recommendations from different retailers are importantly contributed to non-compliance behaviors [14, 18, 29].

Some of the needs expressed by CL wearers have not been addressed by practitioners, such as the needs for the introduction of CL-related information resources, new research results, and CL innovations.

To increase the acceptability of a mobile application embedding functionalities that help the wearers to record their information, have self-care, and interact with the providers is required.

In confirmation of this study’s findings, including entering important information such as the CL name, its brand, and other features, a study showed that many CL wearers could not recall the name of their CL and their CL care products [30]. Consistent with this study’s finding, patients in two studies reported that having information about their activities and tracking of their problems and symptoms facilitate self-care behaviors and self-management strategies [31, 32]. Similarly, patients in a study wanted to contact medical staff for questioning to increase their knowledge and reduce concerns [33] and felt that the communication link between medical staff and patients through a mobile application improves health-related quality of life [32]. These needs have not been reported by practitioners. This is because of difficulty and time-consuming of the task. It is not possible to perform these kinds of services due to the clinic's workload, unless required workforce and financial resources are assigned.

In alignment with our results, Shorey et al. indicated that patients need personalized responses tailored to their condition and symptoms, and online forums provided a clearer and more individualized answer compared with the standard answers found on the web that are usually vague and generic. According to them, finding specific information among questions and comments was difficult and time-consuming. Patients suggested that there should be content sorting and notifications to questions due to burdensome checks of questions repeatedly. Moreover, the patients mentioned that experiences sharing with other people who are undergoing similar encounters could help them understand the issues they were facing and make them feel better about themselves. Based on their study, the patients explained that medical staff should correct the information shared about the experiences to prevent misunderstanding [34].

In agreement with our findings, multiple studies have reported that valuable capabilities include reminders and user tracking assist in personal care and self-handling [31–33]. In a study, Watkins et al. explained that reminders motivate patients to adhere to their treatment and remember their medication and clinical appointment [35], which can minimize the dropout rate in the health care of patients [36]. Although Chong et al. stated that there were concerns about ignoring messages and notifications, and they suggested that messages should be provided in the picture or video form [37] or customized based on their personal preferences and sent to them using their own words to avoid this issue [38].

With regards to the mobile application features subcategories, patients felt that attention should be paid to the structure and interface of mobile application and methods of information presentation. These features play an important role in the acceptance of mobile applications by real users. A great content package alone cannot help increase CL wearers’ knowledge, awareness, and compliance. Coincident with our result, participants in the study of Sewitch et al. described that a sophisticated design would reduce mobile application usage. They suggested utilizing minimal data entry into the mobile application using selection controls such as checkboxes in the user interface design [33]. This is highlighted by Mansouri et al. who mentioned that mobile applications will be acceptable and enhance learning if they are designed to be user-friendly [39]. In line with the current study, different studies have highlighted various features in the development of mobile applications such as easy-to-use and simple design [38], approved information by medical staff [32], links to reliable websites and information resources [35], and a mix of educational materials like video, images, voice, and text [34, 39–41]. Similarly, Rivera et al. reported that participants wanted less information to be provided in the form of text and for there to be minimal reading [31]. Chong et al. described that illustrations and animations stimulate learning in participants and increase information retention [37]. In addition, some studies recommended that training of CL wearers should be accompanied by simple explanations and minimum use of jargon [5, 18] and in order to achieve the greatest benefit for them, training should be conveyed via multimedia such as video, internet resources, or a pamphlet with illustrations [5, 14].

New and old CL wearers generally have common needs. Although, it may not possible to cover the information needs of all CL wearers in an application, it is possible to answer the most common needs of the CL wearers. They may also benefit from the application in various forms. CL wearers who are new to the CL issue, may pay more attention to educational needs with regards to CL care, hygiene, and complications, and also getting to know the service centers and famous brands of lenses. Old CL wearers, according to the experiences they have gained in using the lens, in addition to training and information, may also pay attention to a set of functionalities such as reminders, recording information, asking questions, and commenting.

This study’s findings indicated core components of a mobile application for better CL management and care in CL wearers by involving CL wearers and practitioners to ensure that the end product meets the requirements and expectations of the users. These findings can assist mobile application developers to design a mobile application for CL wearers that would be CL wearers’ friendly and practitioners approved and, policymakers to invest in the development of interventions for more support of CL wearers.

### Limitations

The present study was based upon CL wearers and practitioners who were from three different CL clinics in Iran. While these participants may not be representative of all CL wearers’ requirements because of differences in culture and circumstances in each community, they did provide some of the first information related to creating a CL education application. Likewise, while this study focused on Persian-speaking CL wearers and practitioners, CL wearers face similar issues around the world, and the information gained from this study may be applicable to patients of all languages. This study was lastly only conducted in a hospital setting and because of this, the information gained from the current study may not fully represent other care settings such as private practices or corporate practices. Thus, a future study based upon this work should be conducted to determine if differences exist between different settings regarding patients' needs and requirements.

## Conclusions

Despite many innovations in CL use and care products over the years, CL wearers still face challenges such as obtaining adequate CL knowledge and product awareness. This information is important because it could help patients avoid developing CL complications. The data from the current study provides a clear path forward for the development of an important mobile application for educating all CL wearers about all of the key aspects of CLs and their care. The next steps of this research include the development of the mobile application and testing it in a representative population with the ultimate goal of all practitioners being able to prescribe the mobile application in the clinical setting. The accomplishment of this goal will not only allow practitioners to more easily educate their patients, but it will also make all CL wears safer.

## Supplementary Information

**Additional file 1.** COREQ Checklist.

**Additional file 2.** Example quotations of study participants.

## Data Availability

The datasets used and/or analyzed during the current study are available from the corresponding author on reasonable request.

## Abbreviations

CL: Contact lens
IT: Information Technology
UCD: User-Centered Design
RGP: Rigid gas permeable
PHR: Personal health record
COREQ: Consolidated Criteria for Reporting Qualitative Research
