# Supplementary material for: Identifying the content, functionalities, and features of a mobile application for contact lens wearers
Source: BMC Med Inform Decis Mak. 2022 Jun 21;22:164. doi: 10.1186/s12911-022-01910-w (PMC9215046; doi:10.1186/s12911-022-01910-w)
Supplement: Supplementary file 2 — Additional file 2. Example quotations of study participants. [file 12911_2022_1910_MOESM2_ESM.docx]

| Table 5. Interview excerpts supporting key factors related to ‘Mobile application content‘ category | | | |  |
| --- | --- | --- | --- | --- |
| Subcategories | Sub-subcategories | | Example of interview excerpts |  |
| 1. Advice and precaution | 1.Basic information for CL wearers *** | | I need information on the type of lens, how to use the lens, why I am using it, how the lens works and interacts with the eye, how to prepare for the visit, and the necessary examinations to prescribe the lens. When I want more information about CL, practitioners are always busy, making it difficult to ask additional questions about CL. (CL wearer, male, 27 years old) |  |
|  | 2.CL hygiene information*** | | CL wearers should be given information about lens hygiene, including how to wash and maintain the CL, as well as a description of the risks associated with not adhering to lens hygiene. (Optometrist, female, 8 years of work experience in CL Clinic) |  |
|  | 3.CL care information*** | | CL wearers should take precautions such as not wearing the lens when sleeping, not washing lens with tap water, and only cleaning the lens with special lens solutions. CL wearers are recommended to wear sunglasses, and not to smoke and come into contact with smoke, avoid exposing lens to the elements such as wind and soil, not to place it near a fire or in a hot environment, and avoid using a hair dryer. (Ophthalmologist, male, 37 years of work experience in CL Clinic) |  |
|  | 4.CL complications information*** | | My eyes turned red after using the lens. The lens was annoying. I did not use the lens because I was concerned. I looked for information on various websites. After prescribing the CL, practitioners should explain the complications to CL wearers. (CL wearer, male, 39 years old) |  |
|  | 5.Providing instructions for CL wearers for using the CL** | | Provide CL wearers with clear and concise instructions on how to insert and remove CL, as well as lens care, hygiene, and maintenance. (Ophthalmologist, female, 2 years of work experience in CL Clinic) |  |
|  | 6.Presenting the results of new research regarding safety in using the CL* | | Knowing the results of research such as newly designed lenses, new training tips, solutions to problems such as discomfort and complications of the lens can be very helpful and give us a good feeling and more motivation to use the lens. (CL wearer, male, 27 years old) |  |
| 2.Training | 1.Training of CL insertion tailored to the type of CL*** | | Different lenses require different techniques for insertion and removal. I wanted to learn how to put my lens on and take it off. The clinic's information and practical training were insufficient. There was nowhere for me to get the training again. (CL wearer, female, 34 years old) |  |
|  | 2.Training of CL removal tailored to the type of CL*** | | First, it was difficult for me to put on and take off the lens. I inserted the lens into my eye and my sister removed it. I searched the internet for a good video that would show me how to put and remove lenses. I could not find a suitable video in Persian. (CL wearer, male, 29 years old) |  |
|  | 3.Training of CL rinsing and disinfecting*** | | CL wearers need information such as how to wash and disinfect the lens, how to maintain it, and how frequently the lens solution should be changed. They should be given training in this area because if the lens is not cleaned and maintained properly, it can cause problems. I put the lens in my eye without first washing it and my eye turned red. I was afraid, so I did not use the lens. I contacted the clinic and they assisted me. (CL wearer, male, 27 years old) |  |
|  | 4.Training of hand washing and disinfecting*** | | We should be given instructions on how to wash and disinfect the hands once we receive the CL. The nails should be short. The final rinsing of the hands or fingers with normal saline is crucial in preventing the lens from coming into contact with tap water. It is necessary to point out the dangers of using only tap water. (CL wearer, female, 26 years old) |  |
|  | 5.Training of CL case rinsing and disinfecting*** | | CL wearers should be aware of how to clean and disinfect the CL case, as well as the importance of replacing it and the risks associated with not doing so. (Optometrist, female, 10 years of work experience in CL Clinic) |  |
|  | 6.Specific training tailored to the target population* | | CL wearers with varying educational levels, age groups, and underlying diseases, all receive the same training. What should I know about children's lenses if I need special training for my baby, such as how to put a lens for him? (CL wearer, male, 3 months of age) ^†^ |  |
| 3. Supplementary information for better CL wearing | 1.Introducing CL accessories*** | | CL wearers should be given complete information about CL accessories such as multipurpose solutions, normal saline serum, CL cases, and the CL inserter remover tool. (Ophthalmologist, female, 2 years of work experience in CL Clinic) |  |
|  | 2.Introducing common CL brands*** | | CL wearers should have access to information about the common brands of CL and lens solutions. "Tell us, what the good brands are?" my friends asked me. I talked to my friends about the lens brand.  Some of my friends used low quality colored CL, resulting in red eyes. I recommended good brands to my friends. “Choose between not putting a lens and putting a good lens” I said. (CL wearer, female, 31 years old) |  |
|  | 3.Introducing CL certified sales centers*** | | Teach CL wearers where to get prescription lenses to help eye care practitioners. Who is qualified to prescribe a lens? Prescribing should be done by professional practitioners and based on the results of specialized examinations. Getting CL from a pharmacy or beauty salon is not correct. A number of soft CL wearers use CL without a practitioner's prescription. (Ophthalmologist, male, 37 years of work experience in CL Clinic) |  |
|  | 4.Introducing information resources* | | When I first received the lens, I was curious to learn more about it. I had questions, so I went to the clinic and asked or called the clinic, when I not find answers on the internet. It should be a resource that provides CL wearers with sufficient information. (CL wearer, female, 30 years old) |  |
|  | 5.Introducing new initiatives in the field of CL* | | Introduce lens innovations such as different CL cases designed for different tastes. Fancy CL cases are fun for kids, and they make it easier to distinguish between left and right lenses. For instance, consider the CL cleaner machine. We do not know much about these products in Iran. We only have regular CL cases here. Awareness of these initiatives gives CL wearers a good sense, a sense that there are people who think about CL wearers problems and provide solutions. (CL wearer, male, 27 years old) |  |
|  | 6.Introducing well known centers with their address*** | | Introduce CL practitioners and inform CL wearers about which practitioners in which cities can help them and then, if necessary, refer them to Tehran for further treatment. As a result, the number of patients who must travel is reduced. Some CL wearers receive training in Tehran, but when they return to their hometown, they forget some of what they learned and want to retrain. (CL wearer, female, 36 years old) |  |
| * Needs described by only CL wearers  ** Needs described by only Practitioners  *** Needs described by CL wearers & Practitioners  † The characteristics are related to the CL wearer but their parents answered the questions. | | | |  |
| Table 6. Interview excerpts supporting key factors related to ‘Mobile application functionalities‘ category | | | | |
| Subcategories | | Sub-subcategories | Example of interview excerpts | |
| 1.Entering information required for self-care | | 1. Entering details of practitioner’s visits (visit date, name of clinic, name of practitioner, medications, instructions...) *** | CL wearers forget which clinic they went to and when they went, when they received the lens and when they changed the lens. CL wearers did not follow the practitioner’s instructions because they had forgotten many of instructions. For example, one of CL wearers received the lens a year ago and claims to have received it six months ago. (Optometrist, female, 30 years of work experience in CL Clinic) | |
|  |  | 2.Entering name and type of CL*** | Many CL wearers are unaware of the type of lens or brand they are using. This information is extremely beneficial to me as a practitioner. (Optometrist, female, 20 years of work experience in CL Clinic) | |
|  |  | 3.Entering CL parameters* | It is useful to understand my lens parameters. I travel frequently and need these parameters to present to a practitioner when I visit a clinic in another city or country. (CL wearer, male, 44 years old) | |
|  |  | 4.Entering manufacturing and expiry dates of CL* | I threw away the CL package the first time I opened it. I did not know that production and expiration dates were important. I would write it down in my notebook later. The application’s ability to record this information is excellent. (CL wearer, female, 42 years old) | |
|  |  | 5.Entering start and end dates of CL use* | I had no idea how long I should use the lens. I had to use my lens for 3 months and then replace it, but I used it for a year. I have to record this information so that I do not use the lens after 3 months. (CL wearer, female, 36 years old) | |
|  |  | 6. Entering the hours of gradual use of the hard CL in the CL habit stage*** | When I got the hard lens, I wrote the lens habit information in a notebook. According to the practitioner's instructions, first two hours, then three hours and...  It is very good if the mobile application has the ability to record the information of the CL habit period. (CL wearer, male, 27 years old) | |
|  |  | 7.Entering CL wearing hours during the day*** | The application must be able to record information such as the number of hours spent using CL because CL wearers frequently forget the information and do not know the answers to the questions about CL use time. (Optometrist, female, 11 years of work experience in CL Clinic) | |
|  |  | 8.Entering the time of day when the CL wearers feel discomfort*** | It is critical to note when the CL wearers feel discomfort and how long it occurs after the lens is inserted. Allow the application to record how much discomfort CL wearers are experiencing. (CL wearer, male, 27 years old) | |
|  |  | 9.Entering dates and times of pain while using the CL* | The application should allow us to keep track of how and when the problems occur. For example, I experienced eye pain 18 days after using the lens, as well as the next day. Practitioners usually asked about these times. (CL wearer, male, 37 years old) | |
|  |  | 10.Entering complications and their occurrence time*** | The time of onset of complication is important; for example, does redness appear immediately after wearing the lens or after a few hours? Each of these times gives us different cues to follow the instructions and make recommendations to the CL wearers. (Optometrist, female, 9 years of work experience in CL Clinic) | |
| 2.Computational capability | | 1.Calculation of CL usage time over the day, week and month*** | When I ask the CL wearers how many hours a day they wear the CL, some of them have no idea. It is important for us to know how many hours a day and how many days a week CL wearers put in CL. CL wearers often reduce CL use hours due to discomfort. (Ophthalmologist, female, 10 years of work experience in CL Clinic) | |
|  |  | 2.Calculation of the time period of CL use and the start of uncomfortable feeling** | When I know discomfort time of the CL wearers, I understand that, for example, 5 hours after starting the CL use, CL wearers feels discomfort, which helps us in the course of treatment and the CL use. (Ophthalmologist, female, 2 years of work experience in CL Clinic) | |
|  |  | 3.Calculation of the total days of pain* | When I arrived at the clinic, an eye care practitioner asked me how long I had been experiencing problems. I also forgot whether it was 7 days or 10 days or...? By recording the start time of the problem, the application can calculate the total number of days. (CL wearer, male, 37 years old) | |
| 3. CL wearers interaction with mobile application | | 1.Asking questions from a practitioner* | I would like to have my questions answered. Include the possibility of questions and answers between CL wearers and practitioners in the application. Even if practitioners do not have the time to answer questions online and individually, they can see and answer the questions every few days. (CL wearer, male, 48 years old) | |
|  |  | 2.Creation of frequently asked questions list* | It is very useful to have a list of frequently asked questions. Questions can be updated on a regular basis and questions with a high number of views can be added to the list of frequently asked questions. (CL wearer, male, 51 years old) | |
|  |  | 3.Rating the practitioner’s answers by CL wearers* | When the questions are answered by practitioners, we can give them a score. This helps us understand how good and effective the answer to the question has been. (CL wearer, female, 28 years old) | |
|  |  | 4.Expressing the concerns, opinions and experiences of CL wearers* | It is extremely beneficial to share our experiences especially for CL wearers who are starting to wear lenses. CL wearers express themselves through writing, and I wish a practitioner or an expert would see and approve the opinions. Maybe my experience is wrong. (CL wearer, female, 4 years old) ^†^ | |
|  |  | 5.Rating the experiences and comments* | When the experiences and opinions are rated, we can distinguish the more valuable ones, and we realize that these were tangible for other CL wearers, and most of them had these conditions. (CL wearer, male, 39 years old) | |
|  |  | 6.Comments on the CL wearers’ input* | I write about my own personal experience. Maybe my experience is wrong. It would be great if other CL wearers or practitioners could read the experience and provide feedback. (CL wearer, female, 4 years old) ^†^ | |
|  |  | 7.Organizing and prioritizing CL wearers’ experiences and comments based on the ratings* | Experiences and comments that received the highest score can be displayed. (CL wearer, female, 42 years old) | |
|  |  | 8.Searching answers and experiences* | Over time, the number of responses and comments from CL wearers will increase. As a result, reading them all will be difficult. I have to spend a lot of time looking for information on a topic, so it has to be searchable. (CL wearer, male, 39 years old) | |
|  |  | 9.Making appointments for next visits* | Include the ability to make appointments, especially for those who live in another city and are far from here. (CL wearer, female, 16 years old) | |
| 4.Mobile application development | | 1.Creation of mobile application in different languages such as English in addition to Persian* | If the application is presented in English in addition to Persian, more CL wearers will use it and more comments and problems will be expressed. CL wearer’s data can be used to develop the next version of application as well as to conduct new research to solve many CL wearers’ problems. (CL wearer, female, 36 years old) | |
|  |  | 2. Mobile application updates according to new needs, suggestions, and feedback* | If the application is not kept updated, CL wearers desire to use it will decrease over time. CL wearer’s requests and suggestions should be considered in order to improve the application. (CL wearer, male, 44 years old) | |
|  |  | 3.Development of the mobile application based on best available evidence** | The application's information and content should be up to date with the latest science and research and reflect the most recent published evidence. (Ophthalmologist, female, 10 years of work experience in CL Clinic) | |
| 5.Reminders and alerts | | 1.Setting up the reminders or alerts times*** | CL wearers usually forget the follow-up, when to consult a practitioner, when to change the CL, and so on. Some of them have a regular lifestyle and forget less, while others do not. The application should include a reminder. (Optometrist, female, 10 years of work experience in CL Clinic) | |
|  |  | 2.Reminders of CL use*** | It is possible to set a reminder in the application to remind CL wearers to wear the CL or to give the application a schedule to remind CL wearers to insert and remove the CL on a scheduled basis. (CL wearer, male, 5 years old) ^†^ | |
|  |  | 3.Reminder for CL and CL case replacement time*** | Entering the CL's end date in the application, and then reminding CL wearers one week before that date that the CL needs to be replaced. CL cases should also be replaced regularly. (CL wearer, female, 34 years old) | |
|  |  | 4.Reminder for visiting practitioner*** | A message or an alert could be used to remind the CL wearers of their visit. CL wearers often forget the time of referral to follow the process of using their lens. (Optometrist, female, 30 years of work experience in CL Clinic) | |
|  |  | 5.Alert for entering CL use information*** | Display notifications informing CL wearers that they can enter the information they have. Most of them do not know the answers to the questions related to their lens and its use. (Optometrist, female, 20 years of work experience in CL Clinic) | |
| * Needs described by only CL wearers  ** Needs described by only Practitioners  *** Needs described by CL wearers & Practitioners | | | | |

† The characteristics are related to the CL wearer but their parents answered the questions.

| Table 7. Interview excerpts supporting key factors related to ‘Mobile application features ‘ category | | |
| --- | --- | --- |
| Subcategories | Sub-subcategories | Example of interview excerpts |
| 1.The structure and user interface of the mobile application | 1.Easy to use* | I use a straightforward and uncomplicated application. If I need to find options and spend a significant amount of time each time I use it, I will exit the application and not use it. (CL wearer, male, 51 years old) |
|  | 2.Short and simple tutorials* | I recommend short tutorials and one-to two-minutes videos in simple language. Long content is tedious and ineffective. (CL wearer, female, 16 years old) |
|  | 3.Attractive user interface* | If I want an application, it must have additional features and value as well as an interesting design that I tend to use. I will not make use of it by only providing a list of information. (CL wearer, male, 24 years old) |
|  | 4.Correct and reliable information*** | For me to use an application, it must contain complete and valid information. Every piece of content and application cannot be used and trusted. It must be a valuable and trustworthy application. (CL wearer, female, 26 years old) |
|  | 5.In agreement with the culture* | Educational videos should be made based on the conditions of our community. For example, someone demonstrates how to insert and remove CL by putting a CL in her/his eye and also explains the relevant points. (CL wearer, male, 8 years old) ^†^ |
| 2.Information presentation methods | 1.Text* | The clinic gives us a brochure, but if our vision is poor, we cannot read it. One person is old, the other is illiterate, and both need someone to read the brochure to them. (CL wearer, male, 29 years old) |
|  | 2. Video* | It would be great if you could present the clinic's training as a video in the application. The film is easier to understand. Put different videos, for example, one for the CL wearers to put the lens, one for the person who wants to put the lens for another person, and one for children to put the lens. Insertion and removal of CL, for example, require video, and text alone is not enough. (CL wearer, male, 3 months of age) **^†^** |
|  | 3. Animation* | Tutorials can be presented as one-minute animations that have a greater impact on teaching and learning. (CL wearer, male, 48 years old) |
|  | 4. Voice* | It would be great if the information provided to CL wearers is accompanied by audio. The pamphlet difficult to read for some CL wearers and it contains a lot of words that make it difficult to understand. The audio is very helpful and it is even better because it is spoken in slang. (CL wearer, male, 37 years old) |
| * Needs described by only CL wearers  ** Needs described by only Practitioners  *** Needs described by CL wearers & Practitioners  † The characteristics are related to the CL wearer but their parents answered the questions. | | |
